# 11-(3-Chloro-2-hydroxy­prop­oxy)-2,3,9-trimethoxy­chromeno[3,4-*b*]chromen-12(6*H*)-one

**DOI:** 10.1107/S1600536809018595

**Published:** 2009-05-23

**Authors:** Supranee Sangthong, Thapong Teerawatananond, Chuttree Phurut, Nattaya Ngamrojanavanich, Nongnuj Muangsin

**Affiliations:** a Biotechnology Programme, Research Centre for Bioorganic Chemistry, Faculty of Science, Chulalongkorn University, Pathumwan, Bangkok 10330, Thailand; b Research Centre for Bioorganic Chemistry, Department of Chemistry, Faculty of Science, Chulalongkorn University, Pathumwan, Bangkok 10330, Thailand

## Abstract

In the title compound, C_22_H_21_ClO_8_, the rotenoid core is nearly planar (r.m.s. deviation 0.114 Å), with the largest deviations from the least-squares plane being 0.286 (3) and 0.274 (2) Å. An inter­molecular O—H⋯O hydrogen bond links two mol­ecules into a centrosymmetric dimer having an *R*_2_^2^(18) ring motif.

## Related literature

For a related structure, see: Roengsumran *et al.* (2003 ▶).
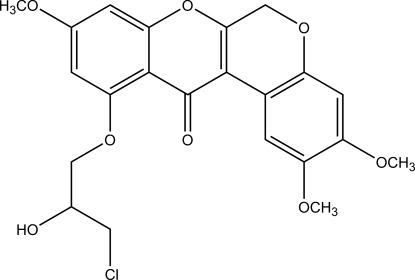


## Experimental

### 

#### Crystal data

C_22_H_21_ClO_8_
*M* *_r_* = 448.84
Triclinic, 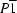

*a* = 7.1534 (4) Å
*b* = 11.7904 (6) Å
*c* = 12.7661 (7) Å
α = 76.901 (3)°
β = 86.991 (3)°
γ = 74.455 (3)°
*V* = 1010.30 (9) Å^3^
*Z* = 2
Mo *K*α radiation
μ = 0.24 mm^−1^
*T* = 293 K
0.30 × 0.24 × 0.20 mm

#### Data collection

Bruker SMART APEXII diffractometer
Absorption correction: none
14308 measured reflections
4517 independent reflections
2879 reflections with *I* > 2σ(*I*)
*R*_int_ = 0.027

#### Refinement

*R*[*F* ^2^ > 2σ(*F* ^2^)] = 0.080
*wR*(*F* ^2^) = 0.268
*S* = 1.05
4517 reflections
272 parameters
7 restraints
H-atom parameters constrained
Δρ_max_ = 0.82 e Å^−3^
Δρ_min_ = −0.91 e Å^−3^

### 

Data collection: *APEX2* (Bruker, 2005 ▶); cell refinement: *SAINT* (Bruker, 2005 ▶); data reduction: *SAINT*; program(s) used to solve structure: *SHELXS97* (Sheldrick, 2008 ▶); program(s) used to refine structure: *SHELXL97* (Sheldrick, 2008 ▶); molecular graphics: *ORTEP-3* (Farrugia, 1997 ▶); software used to prepare material for publication: *publCIF* (Westrip, 2009 ▶).

## Supplementary Material

Crystal structure: contains datablocks I, global. DOI: 10.1107/S1600536809018595/ng2583sup1.cif

Structure factors: contains datablocks I. DOI: 10.1107/S1600536809018595/ng2583Isup2.hkl

Additional supplementary materials:  crystallographic information; 3D view; checkCIF report

## Figures and Tables

**Table 1:** Hydrogen-bond geometry (Å, °)

*D*—H⋯*A*	*D*—H	H⋯*A*	*D*⋯*A*	*D*—H⋯*A*
O8′—H8′⋯O8′^i^	0.82	2.2	2.81 (2)	132
O8′—H8′⋯O7^i^	0.82	2.54	3.297 (10)	154
O8—H8⋯O3^i^	0.82	1.92	2.735 (6)	169

Symmetry code: (i) 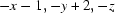.

**Fig. 1.**
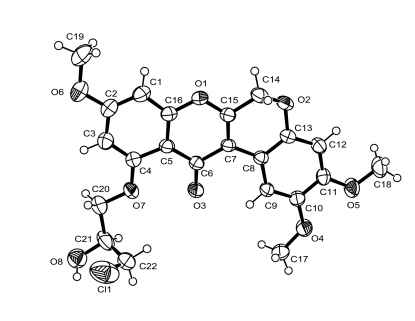
The molecular structure of the title compound, with disordered omitted. Displacement ellipsoids are drawn at 50% probability level.

**Fig. 2.**
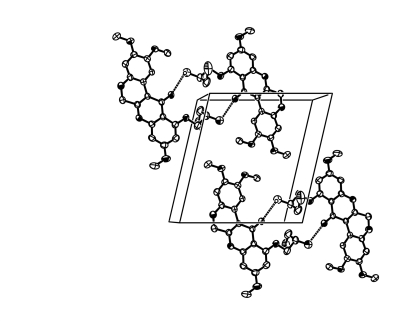
Packing diagram of A hydrogen bonded dimer of the title compound. Hydrogen bonds are shown as dashed lines.

## supplementary crystallographic information

### Comment

6-Deoxyclitoriacetal, extracted from the roots of *Stemona Collinsae* Craib, showed strong cytotoxic activity against various human carcinoma (Roengsumran *et al.*, 2003). In order to enhance its cytotoxic activities, the title compound was synthesized and its crystal structure was reported herein.

The bond lengths and angles in the molecules (Fig. 1) are within normal ranges and are comparable to a closely related structure (Roengsumran *et al.*, 2003). The rotenoid core is nearly flattened with the largest deviations from the least-squares plane of 0.286 (3) at C14 and -0.274 (2) at O2.

In the crystal structure, interatomic OH···O hydrogen bonds link the molecules into centrosymmetric dimers, forming *R*_2_^2^(18) ring motifs, in which they may help in stabilize the crystal structure (Table 1).

### Experimental

To the reaction mixture of 3-(4,5-dimethoxy-2-oxiranylmethoxy-phenyl)-3-hydroxy-7-methoxy-2-methyl-5-oxiranylmethoxy-chroman-4-one (20 mg, 0.046 mmol) in 3 ml of aqueous ethyl acetate was added 1*M* HCl (3 ml). The mixture solution was stirred for 30 min at room temperature. The reaction mixture was evaporated to remove solvent under reduced pressure. The residue was washed with water. Organic layer was extracted with dichloromethane and dried over MgSO_4_. Solvent was removed under reduced pressure and the crude product was purified by silica gel column chromatography with dichloromethane:ethyl acetate:dichloromethane (2:1:2, *v*/*v*) as elutent to give the title compound as yellow crystal (70% yield).

### Refinement

All non-H atoms were anisotropically refined. The O8 atom is disordered over two positions with site occupancies of 0.710 (5) and 0.290 (5). Restraints were used (SADI, *DFIX* and ISOR). The H atoms were positioned geometrically and refined using a riding model, with C—H = 0.93 Å (aromatic), 0.97 Å (CH_2_), 0.98 Å (CH_3_) and O—H = 0.82 Å, and *U*_iso_(H) = 1.2*U*eq (C_aromatic_), 1.5*U*eq (C_CH2_), 1.5*U*eq (C_CH3_) and 1.2*U*eq (C_O_), respectively.

### Crystal data

**Table:**

C_22_H_21_ClO_8_	*V* = 1010.30 (9) Å^3^
*M**_r_* = 448.84	*Z* = 2
Triclinic, *P*1	*F*(000) = 468
Hall symbol: -P 1	*D*_x_ = 1.475 Mg m^−^^3^
*a* = 7.1534 (4) Å	Mo *K*α radiation, λ = 0.71073 Å
*b* = 11.7904 (6) Å	θ = 1.6–27.4°
*c* = 12.7661 (7) Å	µ = 0.24 mm^−^^1^
α = 76.901 (3)°	*T* = 293 K
β = 86.991 (3)°	Prism, colourless
γ = 74.455 (3)°	0.30 × 0.24 × 0.20 mm

### Data collection

**Table:**

Bruker SMART APEXII diffractometer	*R*_int_ = 0.027
Radiation source: Mo	θ_max_ = 27.4°, θ_min_ = 1.6°
ω scans	*h* = −8→9
14308 measured reflections	*k* = −15→14
4517 independent reflections	*l* = −16→16
2879 reflections with *I* > 2σ(*I*)	

### Refinement

**Table:**

Refinement on *F*^2^	7 restraints
Least-squares matrix: full	H-atom parameters constrained
*R*[*F*^2^ > 2σ(*F*^2^)] = 0.080	*w* = 1/[σ^2^(*F*_o_^2^) + (0.1402*P*)^2^ + 1.0019*P*] where *P* = (*F*_o_^2^ + 2*F*_c_^2^)/3
*wR*(*F*^2^) = 0.268	(Δ/σ)_max_ = 0.058
*S* = 1.05	Δρ_max_ = 0.82 e Å^−^^3^
4517 reflections	Δρ_min_ = −0.91 e Å^−^^3^
272 parameters	

### Special details

**Table:**

Geometry. All e.s.d.'s (except the e.s.d. in the dihedral angle between two l.s. planes) are estimated using the full covariance matrix. The cell e.s.d.'s are taken into account individually in the estimation of e.s.d.'s in distances, angles and torsion angles; correlations between e.s.d.'s in cell parameters are only used when they are defined by crystal symmetry. An approximate (isotropic) treatment of cell e.s.d.'s is used for estimating e.s.d.'s involving l.s. planes.

### Fractional atomic coordinates and isotropic or equivalent isotropic displacement parameters (Å^2^)

**Table:**

	*x*	*y*	*z*	*U*_iso_*/*U*_eq_	Occ. (<1)
Cl1	−0.0091 (2)	0.94031 (14)	0.19387 (18)	0.1035 (7)	
O1	−0.8374 (4)	1.5195 (2)	0.1837 (2)	0.0440 (6)	
O2	−0.9242 (4)	1.7345 (2)	−0.0626 (2)	0.0530 (7)	
O3	−0.6183 (5)	1.2837 (2)	−0.0085 (2)	0.0565 (8)	
O4	−0.5594 (5)	1.5045 (3)	−0.3767 (2)	0.0589 (8)	
O5	−0.7253 (5)	1.7328 (3)	−0.4252 (2)	0.0598 (8)	
O6	−0.7703 (5)	1.2142 (3)	0.5002 (2)	0.0646 (9)	
O7	−0.5510 (5)	1.1103 (2)	0.1704 (2)	0.0566 (8)	
C21	−0.3641 (7)	0.9229 (3)	0.1446 (4)	0.063	
H21	−0.4543	0.9373	0.0849	0.075*	0.710 (5)
H21'	−0.3023	0.839	0.1856	0.075*	0.290 (5)
O8	−0.2980 (6)	0.7966 (4)	0.1808 (4)	0.064*	0.710 (5)
H8	−0.3167	0.7636	0.1337	0.096*	0.710 (5)
O8'	−0.5162 (14)	0.9032 (9)	0.0844 (8)	0.062*	0.290 (5)
H8'	−0.4975	0.9235	0.0198	0.093*	0.290 (5)
C1	−0.8078 (6)	1.3721 (4)	0.3395 (3)	0.0460 (9)	
H1	−0.8647	1.4325	0.376	0.055*	
C2	−0.7492 (6)	1.2532 (4)	0.3927 (3)	0.0475 (9)	
C3	−0.6659 (6)	1.1635 (4)	0.3367 (3)	0.0493 (9)	
H3	−0.6288	1.0833	0.3737	0.059*	
C4	−0.6377 (6)	1.1925 (3)	0.2272 (3)	0.0440 (9)	
C5	−0.6986 (5)	1.3151 (3)	0.1675 (3)	0.0368 (8)	
C6	−0.6830 (5)	1.3541 (3)	0.0510 (3)	0.0384 (8)	
C7	−0.7431 (5)	1.4857 (3)	0.0087 (3)	0.0334 (7)	
C8	−0.7322 (5)	1.5470 (3)	−0.1049 (3)	0.0346 (7)	
C9	−0.6415 (5)	1.4904 (3)	−0.1873 (3)	0.0383 (8)	
H9	−0.5796	1.4084	−0.1704	0.046*	
C10	−0.6422 (6)	1.5539 (3)	−0.2928 (3)	0.0418 (8)	
C11	−0.7324 (6)	1.6778 (3)	−0.3193 (3)	0.0427 (8)	
C12	−0.8198 (5)	1.7351 (3)	−0.2397 (3)	0.0437 (9)	
H12	−0.8786	1.8176	−0.2565	0.052*	
C13	−0.8204 (5)	1.6705 (3)	−0.1351 (3)	0.0392 (8)	
C14	−0.8613 (7)	1.6920 (3)	0.0448 (3)	0.0506 (10)	
H14A	−0.963	1.7257	0.091	0.061*	
H14B	−0.7485	1.7195	0.0543	0.061*	
C15	−0.8106 (5)	1.5572 (3)	0.0779 (3)	0.0386 (8)	
C16	−0.7789 (5)	1.3987 (3)	0.2294 (3)	0.0389 (8)	
C17	−0.4440 (7)	1.3837 (4)	−0.3532 (3)	0.0577 (11)	
H17A	−0.3951	1.3606	−0.4189	0.087*	
H17B	−0.5212	1.3315	−0.3172	0.087*	
H17C	−0.3373	1.3769	−0.3075	0.087*	
C18	−0.8071 (8)	1.8595 (4)	−0.4555 (4)	0.0658 (13)	
H18A	−0.7916	1.8867	−0.5315	0.099*	
H18B	−0.7423	1.8994	−0.4172	0.099*	
H18C	−0.9427	1.878	−0.4384	0.099*	
C19	−0.8712 (9)	1.3025 (5)	0.5581 (4)	0.0774 (15)	
H19A	−0.8778	1.2646	0.6326	0.116*	
H19B	−1.0002	1.3384	0.5293	0.116*	
H19C	−0.8034	1.3639	0.5512	0.116*	
C20	−0.4775 (7)	0.9901 (3)	0.2280 (4)	0.0574 (11)	
H20A	−0.5823	0.9553	0.2579	0.069*	
H20B	−0.3926	0.9867	0.2861	0.069*	
C22	−0.1968 (9)	0.9713 (4)	0.0981 (5)	0.0854 (19)	
H22A	−0.2427	1.0579	0.0714	0.102*	
H22B	−0.1453	0.9357	0.0377	0.102*	

### Atomic displacement parameters (Å^2^)

**Table:**

	*U*^11^	*U*^22^	*U*^33^	*U*^12^	*U*^13^	*U*^23^
Cl1	0.0831 (11)	0.0732 (10)	0.1646 (18)	−0.0261 (8)	0.0238 (10)	−0.0457 (11)
O1	0.0532 (15)	0.0392 (14)	0.0383 (14)	−0.0067 (11)	0.0102 (11)	−0.0147 (11)
O2	0.0622 (18)	0.0374 (14)	0.0484 (16)	0.0066 (12)	0.0055 (13)	−0.0121 (12)
O3	0.087 (2)	0.0332 (14)	0.0452 (16)	−0.0064 (13)	0.0152 (14)	−0.0156 (12)
O4	0.093 (2)	0.0470 (16)	0.0332 (14)	−0.0100 (15)	0.0080 (13)	−0.0128 (12)
O5	0.084 (2)	0.0487 (16)	0.0385 (15)	−0.0118 (15)	−0.0030 (14)	−0.0003 (12)
O6	0.078 (2)	0.072 (2)	0.0392 (16)	−0.0197 (16)	0.0067 (14)	−0.0040 (15)
O7	0.078 (2)	0.0320 (14)	0.0488 (16)	−0.0001 (13)	0.0084 (14)	−0.0057 (12)
C21	0.085	0.033	0.063	0	−0.011	−0.011
C1	0.050 (2)	0.053 (2)	0.040 (2)	−0.0170 (17)	0.0080 (16)	−0.0177 (17)
C2	0.048 (2)	0.057 (2)	0.040 (2)	−0.0202 (18)	0.0058 (16)	−0.0085 (18)
C3	0.048 (2)	0.045 (2)	0.051 (2)	−0.0122 (17)	0.0055 (17)	−0.0033 (18)
C4	0.046 (2)	0.0394 (19)	0.044 (2)	−0.0087 (15)	0.0068 (15)	−0.0075 (16)
C5	0.0376 (18)	0.0336 (17)	0.0400 (19)	−0.0086 (13)	0.0051 (14)	−0.0117 (14)
C6	0.0443 (19)	0.0310 (17)	0.0410 (19)	−0.0091 (14)	0.0063 (15)	−0.0124 (15)
C7	0.0331 (17)	0.0309 (16)	0.0372 (17)	−0.0072 (12)	0.0040 (13)	−0.0121 (14)
C8	0.0335 (17)	0.0331 (17)	0.0386 (18)	−0.0091 (13)	0.0018 (13)	−0.0107 (14)
C9	0.047 (2)	0.0303 (16)	0.0366 (18)	−0.0076 (14)	0.0031 (14)	−0.0095 (14)
C10	0.049 (2)	0.0412 (19)	0.0370 (19)	−0.0118 (15)	0.0010 (15)	−0.0116 (15)
C11	0.049 (2)	0.0403 (19)	0.0364 (19)	−0.0126 (16)	−0.0054 (15)	−0.0024 (15)
C12	0.046 (2)	0.0349 (18)	0.046 (2)	−0.0046 (15)	−0.0035 (16)	−0.0057 (16)
C13	0.0401 (19)	0.0341 (18)	0.0427 (19)	−0.0053 (14)	0.0017 (14)	−0.0126 (15)
C14	0.062 (3)	0.0362 (19)	0.049 (2)	−0.0019 (17)	0.0034 (18)	−0.0157 (17)
C15	0.0379 (18)	0.0354 (18)	0.0417 (19)	−0.0064 (14)	0.0038 (14)	−0.0114 (15)
C16	0.0381 (18)	0.0401 (19)	0.0404 (19)	−0.0105 (14)	0.0043 (14)	−0.0135 (15)
C17	0.079 (3)	0.051 (2)	0.044 (2)	−0.013 (2)	0.015 (2)	−0.0207 (19)
C18	0.086 (3)	0.050 (2)	0.052 (3)	−0.013 (2)	−0.007 (2)	0.006 (2)
C19	0.102 (4)	0.093 (4)	0.038 (2)	−0.025 (3)	0.011 (2)	−0.018 (2)
C20	0.065 (3)	0.039 (2)	0.061 (3)	−0.0100 (18)	0.003 (2)	−0.0015 (19)
C22	0.117 (5)	0.037 (2)	0.079 (4)	0.004 (2)	0.039 (3)	−0.007 (2)

### Geometric parameters (Å, °)

**Table:**

Cl1—C22	1.772 (7)	C5—C16	1.389 (5)
O1—C15	1.343 (4)	C5—C6	1.462 (5)
O1—C16	1.369 (4)	C6—C7	1.473 (5)
O2—C13	1.386 (4)	C7—C15	1.342 (5)
O2—C14	1.402 (5)	C7—C8	1.474 (5)
O3—C6	1.233 (4)	C8—C13	1.394 (5)
O4—C10	1.367 (5)	C8—C9	1.410 (5)
O4—C17	1.415 (5)	C9—C10	1.383 (5)
O5—C11	1.366 (4)	C9—H9	0.93
O5—C18	1.420 (5)	C10—C11	1.400 (5)
O6—C2	1.359 (5)	C11—C12	1.379 (6)
O6—C19	1.428 (6)	C12—C13	1.379 (5)
O7—C4	1.342 (5)	C12—H12	0.93
O7—C20	1.412 (5)	C14—C15	1.496 (5)
C21—O8	1.410 (5)	C14—H14A	0.97
C21—O8'	1.460 (7)	C14—H14B	0.97
C21—C22	1.500 (8)	C17—H17A	0.96
C21—C20	1.538 (6)	C17—H17B	0.96
C21—H21	0.98	C17—H17C	0.96
C21—H21'	1.0012	C18—H18A	0.96
O8—H21'	0.5101	C18—H18B	0.96
O8—H8	0.82	C18—H18C	0.96
O8'—H8'	0.82	C19—H19A	0.96
C1—C2	1.373 (6)	C19—H19B	0.96
C1—C16	1.386 (5)	C19—H19C	0.96
C1—H1	0.93	C20—H20A	0.97
C2—C3	1.394 (6)	C20—H20B	0.97
C3—C4	1.379 (6)	C22—H22A	0.97
C3—H3	0.93	C22—H22B	0.97
C4—C5	1.435 (5)		
			
C15—O1—C16	119.0 (3)	O4—C10—C11	115.4 (3)
C13—O2—C14	115.8 (3)	C9—C10—C11	119.8 (3)
C10—O4—C17	118.1 (3)	O5—C11—C12	124.9 (3)
C11—O5—C18	117.9 (3)	O5—C11—C10	115.7 (3)
C2—O6—C19	117.0 (4)	C12—C11—C10	119.4 (3)
C4—O7—C20	117.3 (3)	C13—C12—C11	120.2 (3)
O8—C21—O8'	88.0 (5)	C13—C12—H12	119.9
O8—C21—C22	109.0 (4)	C11—C12—H12	119.9
O8'—C21—C22	126.5 (6)	C12—C13—O2	115.7 (3)
O8—C21—C20	115.0 (4)	C12—C13—C8	122.5 (3)
O8'—C21—C20	103.5 (5)	O2—C13—C8	121.7 (3)
C22—C21—C20	112.9 (4)	O2—C14—C15	112.2 (3)
O8—C21—H21	106.4	O2—C14—H14A	109.2
O8'—C21—H21	22.8	C15—C14—H14A	109.2
C22—C21—H21	106.4	O2—C14—H14B	109.2
C20—C21—H21	106.4	C15—C14—H14B	109.2
O8—C21—H21'	14.8	H14A—C14—H14B	107.9
O8'—C21—H21'	101.7	O1—C15—C7	125.7 (3)
C22—C21—H21'	104.5	O1—C15—C14	111.6 (3)
C20—C21—H21'	105.8	C7—C15—C14	122.7 (3)
H21—C21—H21'	121	O1—C16—C5	121.0 (3)
C21—O8—H21'	30	O1—C16—C1	113.4 (3)
C21—O8—H8	109.5	C5—C16—C1	125.6 (3)
H21'—O8—H8	138.7	O4—C17—H17A	109.5
C21—O8'—H8'	109.5	O4—C17—H17B	109.5
C2—C1—C16	117.5 (4)	H17A—C17—H17B	109.5
C2—C1—H1	121.2	O4—C17—H17C	109.5
C16—C1—H1	121.2	H17A—C17—H17C	109.5
O6—C2—C1	123.8 (4)	H17B—C17—H17C	109.5
O6—C2—C3	115.7 (4)	O5—C18—H18A	109.5
C1—C2—C3	120.6 (4)	O5—C18—H18B	109.5
C4—C3—C2	120.8 (4)	H18A—C18—H18B	109.5
C4—C3—H3	119.6	O5—C18—H18C	109.5
C2—C3—H3	119.6	H18A—C18—H18C	109.5
O7—C4—C3	123.1 (3)	H18B—C18—H18C	109.5
O7—C4—C5	116.0 (3)	O6—C19—H19A	109.5
C3—C4—C5	120.9 (3)	O6—C19—H19B	109.5
C16—C5—C4	114.6 (3)	H19A—C19—H19B	109.5
C16—C5—C6	120.4 (3)	O6—C19—H19C	109.5
C4—C5—C6	125.0 (3)	H19A—C19—H19C	109.5
O3—C6—C5	123.4 (3)	H19B—C19—H19C	109.5
O3—C6—C7	121.5 (3)	O7—C20—C21	104.9 (3)
C5—C6—C7	115.1 (3)	O7—C20—H20A	110.8
C15—C7—C8	116.3 (3)	C21—C20—H20A	110.8
C15—C7—C6	118.4 (3)	O7—C20—H20B	110.8
C8—C7—C6	125.2 (3)	C21—C20—H20B	110.8
C13—C8—C9	116.4 (3)	H20A—C20—H20B	108.8
C13—C8—C7	118.4 (3)	C21—C22—Cl1	112.2 (4)
C9—C8—C7	125.2 (3)	C21—C22—H22A	109.2
C10—C9—C8	121.7 (3)	Cl1—C22—H22A	109.2
C10—C9—H9	119.1	C21—C22—H22B	109.2
C8—C9—H9	119.1	Cl1—C22—H22B	109.2
O4—C10—C9	124.7 (3)	H22A—C22—H22B	107.9

### Hydrogen-bond geometry (Å, °)

**Table:**

*D*—H···*A*	*D*—H	H···*A*	*D*···*A*	*D*—H···*A*
O8'—H8'···O8'^i^	0.82	2.2	2.81 (2)	132
O8'—H8'···O7^i^	0.82	2.54	3.297 (10)	154
O8—H8···O3^i^	0.82	1.92	2.735 (6)	169

Symmetry codes: (i) −*x*−1, −*y*+2, −*z*.
